# Diagnosing Cystic Fibrosis in the 21st Century—A Complex and Challenging Task

**DOI:** 10.3390/diagnostics14070763

**Published:** 2024-04-03

**Authors:** Dana-Teodora Anton-Păduraru, Alice Nicoleta Azoicăi, Felicia Trofin, Dana Elena Mîndru, Alina Mariela Murgu, Ana Simona Bocec, Codruța Olimpiada Iliescu Halițchi, Carmen Iulia Ciongradi, Ioan Sȃrbu, Maria Liliana Iliescu

**Affiliations:** 1 Department of Mother and Child Medicine, “Grigore T. Popa” University of Medicine and Pharmacy, 700115 Iaṣi, Romania; dana.anton@umfiasi.ro (D.-T.A.-P.); alice.azoicai@umfiasi.ro (A.N.A.); mindru.dana@umfiasi.ro (D.E.M.); alina.murgu@umfiasi.ro (A.M.M.); ana-simona.drochioi@umfiasi.ro (A.S.B.); olimpiada.iliescu@umfiasi.ro (C.O.I.H.); 2 “Sf.Maria” Children Emergency Hospital, 700309 Iaṣi, Romania; carmen.ciongradi@umfiasi.ro (C.I.C.); sarbu.ioan@umfiasi.ro (I.S.); 3 Department of Preventive Medicine and Interdisciplinarity—Microbiology, “Grigore T. Popa” University of Medicine and Pharmacy, 700115 Iaṣi, Romania; 4 2nd Department of Surgery, Pediatric Surgery and Orthopedics, “Grigore T. Popa” University of Medicine and Pharmacy, 700115 Iaṣi, Romania; 5 Department of Preventive Medicine and Interdisciplinarity—Public Health and Health Management, “Grigore T. Popa” University of Medicine and Pharmacy, 700115 Iaṣi, Romania; maria.iliescu@umfiasi.ro

**Keywords:** cystic fibrosis, children, diagnostic, neonatal screening, sweat test, genetic

## Abstract

Cystic fibrosis (CF) is a chronic and potentially life-threatening condition, wherein timely diagnosis assumes paramount significance for the prompt initiation of therapeutic interventions, thereby ameliorating pulmonary function, addressing nutritional deficits, averting complications, mitigating morbidity, and ultimately enhancing the quality of life and extending longevity. This review aims to amalgamate existing knowledge to provide a comprehensive appraisal of contemporary diagnostic modalities pertinent to CF in the 21st century. Deliberations encompass discrete delineations of each diagnostic modality and the elucidation of potential diagnostic quandaries encountered in select instances, as well as the delineation of genotype–phenotype correlations germane to genetic counseling endeavors. The synthesis underscores that, notwithstanding the availability and strides in diagnostic methodologies, including genetic assays, the sweat test (ST) retains its position as the preeminent diagnostic standard for CF, serving as a robust surrogate for CFTR functionality. Prospective clinical investigations in the realm of CF should be orchestrated with the objective of discerning novel diagnostic modalities endowed with heightened specificity and sensitivity.

## 1. Introduction

Cystic fibrosis (CF) is as a rare autosomal recessive condition characterized by a chronic trajectory, typified by exocrine pancreatic insufficiency, chronic pulmonary afflictions, and augmented concentrations of chlorine and sodium in sweat [1,2,3,4]. It ranks as the second most life-threatening monogenic disorder subsequent to sickle cell disease [5]. Within CF pathology, sweat secretion remains unaltered by cholinergic agents, yet the secretion elicited via beta-adrenergic pathways is contingent upon cystic fibrosis transmembrane conductance regulator (CFTR) and is diminished or absent within the sweat glands of CF patients. Heterozygotes manifest a sweat rate half that of normative subjects, while CF individuals with pancreatic insufficiency exhibit a null sweat rate [6]. Clinical manifestations vary in severity, determined by the interplay of three factors: the inherent defect within the responsible gene, the milieu in which the defective gene operates, and environmental influences [7,8]. Indications of CF diagnosis are underscored by distinctive symptoms, familial CF history, and positive neonatal screening results [2]. The establishment of CF diagnosis bears implications and reverberations for the patient and familial milieu [9]. Timely diagnosis assumes paramount significance in expeditiously commencing treatment, thereby fostering enhancements in pulmonary function and nutritional status, curtailing hospitalizations and morbidity rates, and ultimately augmenting the quality of life and extending life expectancy [10,11]. However, due to the fact that neonatal screening is not mandatory worldwide, diagnostic delays are prevalent, with the disease frequently being identified at an advanced stage [5]. This could be attributed to either a failure to identify the urgent warning signs necessitating immediate investigation for CF, or the inability to conduct a timely sweat test due to budget constraints or insufficient medical personnel.

The aims of our review encompass the synthesis of extant knowledge with the intent of furnishing a comprehensive delineation of CF diagnostic prospects in the 21st century, including the presentation of data pertinent to individual diagnostic modalities alongside the potential diagnostic challenges encountered in select scenarios.

## 2. Literature Search

We performed an electronic search of the literature in PubMed, EMBASE, and Google Academic, using the following search terms: “cystic fibrosis”, “children”, “diagnostic”, “neonatal screening”, “sweat test”, “genetic mutation”, and “nasal potential difference”. We assessed all included articles depending on the suitability of the methods used to check the hypothesis, the key results, the interpretation of the results, the quality of the results, and the relevance of the conclusions. All articles that did not match these criteria were excluded from the study as well as those written in languages other than English or those that had only the abstract available. When assessing the articles, we also identified supplementary references that matched our criteria, and we performed a manual search in the reference list of each article retrieved. Finally, we synthetized the results from all 180 articles included in references.

**Figure 1:**
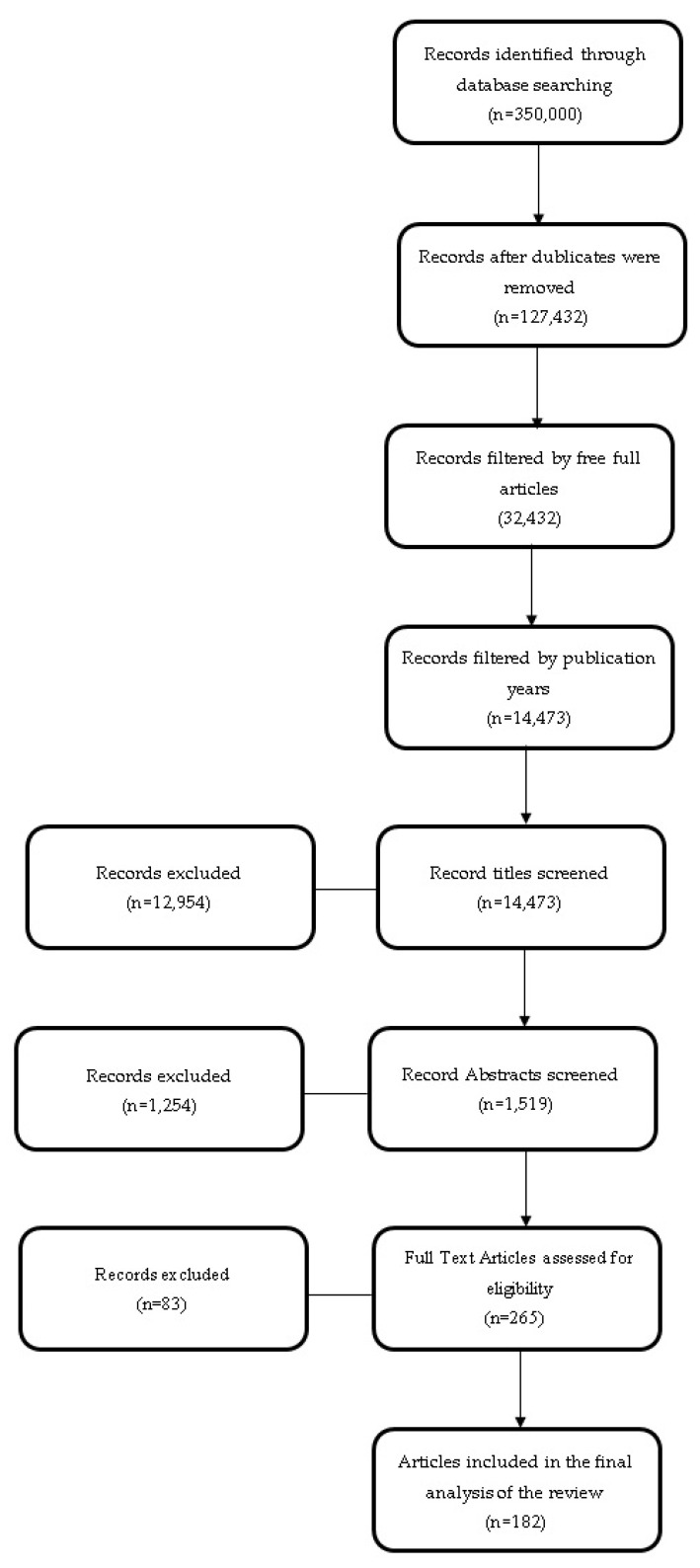
The review flowchart.

The findings from the articles cited in the references were consolidated and organized in a systematic manner to create the basis of this review article. A well-organized framework was meticulously developed to structure pertinent information. Figure 1 illustrates the step-by-step flow of information during the review, visually representing the identification, inclusion, and exclusion of records.

## 3. Results

### 3.1. Diagnostic Approaches in CF

#### 3.1.1. Prenatal Diagnostic Techniques

The prenatal diagnostic process involves chorionic villus sampling during the first trimester or amniocentesis in the second and third trimesters of gestation [12]. Nonetheless, the preference leans towards non-invasive prenatal diagnostic methods [13]. Several researchers have highlighted the importance of mitigating the risk of spontaneous abortion associated with chorionic villus sampling, improving test accuracy, and enabling early diagnostic opportunities as pivotal factors in the development of non-invasive prenatal testing (NIPT) through DNA analysis or the examination of circulating trophoblasts in maternal blood [14,15,16]. Initially reliant on detecting or excluding the paternal allele from maternal plasma, the NIPT approach, employing fetal cell-free DNA, serves its purpose only when parents harbor different CF gene variants, rendering it unsuitable for CF screening within populations exhibiting significant mutational homogeneity. Furthermore, identifying the paternal variant allele necessitates subsequent invasive procedures to ascertain the fetal status regarding the maternal variant allele [17].

Around the 10–14 week gestational mark, intact circulating trophoblasts, constituting the pinnacle of cell-based NIPT, can be consistently isolated, allowing for the extraction of placental DNA without recourse to maternal DNA sampling and analysis [18]. This method holds promise for prenatal screening endeavors as direct variant analysis can be conducted without the need for additional genetic tests [19,20].

#### 3.1.2. Neonatal Screening

According to Castellani et al. (2009), “the goal of neonatal screening should be to find the greatest proportion of CF-patients as possible with the least number of false positive tests” [21]. The identification of elevated levels of trypsinogen (IRT), a precursor enzyme of trypsin, facilitated the inception of neonatal screening programs, newborn bloodspot screening (NBS), albeit their universal adoption remains incomplete. Although the implementation of the NBS has bolstered the diagnosis of CF cases during the neonatal phase, its diagnostic specificity remains modest, necessitating subsequent verification through sweat testing (ST) and genetic mutation analysis [22,23]. Moreover, false positive or negative outcomes in screening programs may arise due to various factors, such as erroneous testing procedures or diminished trypsinogen levels [12,24].

In instances where neonatal screening yields positive results (>70 ng/mL) but fewer than two CF-causing mutations are identified, recommendations entail undergoing a sweat test or nasal potential difference (NPD) assessment to ascertain CFTR dysfunction [25,26]. The decision to classify such patients as having CF should be meticulously deliberated, incorporating clinical assessment, CFTR functional appraisal, and genetic scrutiny [26].

If neonatal screening indicates positivity, yet the patient lacks indicative disease symptoms, and the sweat test yields either negative results with two CFTR mutations, one of which carries ambiguous phenotypic implications, or equivocal outcomes with 0–1 CF-causing mutations, the individual may be categorized as having CFTR-related metabolic syndrome (CRMS) in the USA or CF screen positive, inconclusive diagnosis (CFSPID) in Europe [27]. Recently, consensus among experts has been reached on an international definition of CRMS/CFSPID, aimed at refining the understanding of the epidemiology, management, and prognosis of these infants and optimizing the outcomes of future investigations focused on these patient cohorts [28].

In such scenarios, repeat sweat testing is imperative, alongside an expanded genetic analysis encompassing sequencing, deletion, and duplication assessments, and CFTR functional evaluations. These patients necessitate periodic monitoring via sputum or hypopharyngeal aspirate cultures, radiological assessments, spirometry, lung clearance index determinations, and fecal elastase assays, with follow-up frequency and duration tailored to individual circumstances. Healthcare providers should identify and communicate this complex situation to families, necessitating ongoing follow-up and, at times, therapeutic interventions if they will develop a CFTR-related disorder or convert to a CF diagnosis [28]. Genetic counseling is indispensable for families with CRMS/CFSPID to comprehend the risk of CF occurrence in subsequent pregnancies if both parents carried CFTR variants that were known to be pathogenic or if different partners are involved [29]. Research exploring the phenotypic expression of infants diagnosed with CFSPID has unveiled decreased levels of immunoreactive trypsinogen (IRT) and sweat chloride compared to confirmed CF cases. In the study conducted by Arrudi-Moreno et al. (2021), CFSPID patients exhibited lower IRT levels (mean 93.53 ng/mL) compared to those with CF (mean 175.38 ng/mL) [30]. Similar findings were reported by the following:

- Ooi et al. (2019), reported a median IRT concentration of 75 ng/mL in CFSPID patients versus 143.8 ng/mL in CF cases [31].
- Castaldo et al. (2020) reported a difference of 71.53 ng/mL versus 136.8 ng/mL [32].
- Gunnett et al. (2023) reported a difference of 66.7 ng/mL versus 158.5 ng/mL [33].

Additionally, sweat test values were lower in CFSPID patients compared to those with CF, observed to be 16.2 mmol/L versus 76.1 mmol/L in Castaldo et al.’s study (2020), and 33.7 mmol/L versus 95 mmol/L in Gunnett et al.’s study (2023) [32,33]. Terlizzi and Dolce (2023) found that CFSPID patients exhibited more variability in sweat test values than CF infants. Specifically, 16.2% out of 37 CFSPID cases had their first sweat test value within the normal range, while 83.8% fell within the intermediate range [34]. These infants exhibit milder symptoms, improved nutritional statuses, and reduced hospitalization rates. Notably, the prevalence of *Pseudomonas aeruginosa* and methicillin-resistant *Staphylococcus aureus* in sputum and broncho-alveolar lavage fluid during a 6–7-year follow-up was low, according Munck et al. (2020) [28]. Furthermore, according to Castaldo et al. (2020), the incidence of *P. aeruginosa* and *S. maltophilia* infections was less common in CFSPID patients compared to those with CF, with rates of 28.7% versus 50% and 12.5% versus 39.1%, respectively [32].

Imaging evaluations, pulmonary function tests, and nutritional status assessments of pediatric patients with ambiguous CF diagnoses reveal higher scores than control groups. It is noteworthy that some patients with uncertain diagnoses were subsequently confirmed with CF following genetic testing. Hence, until the age of 6–7 years, the disparities between confirmed and suspected CF patients necessitate vigilant monitoring and supplementary testing to mitigate the risk of false negative diagnoses [28]. The diagnosis in patients not included in screening is a challenge because the age of onset and the severity of symptoms differ depending on the degree of CFTR dysfunction [26].

#### 3.1.3. Sweat Test (ST)

A. Background Information

CF was initially delineated over eight decades ago, with the term “cystic fibrosis of the pancreas” coined in 1938. Notably, in 1948, diSant Agnese observed the phenomenon of excessive salt loss through sweat in CF patients. Subsequently, the documentation in 1953 of elevated electrolyte levels in sweat prompted the introduction of quantitative sweat testing by Schwachman and Gahm in 1956 [35,36]. This method, regarded as a simple and reproducible diagnostic approach with commendable accuracy, persists as the “gold standard” for CF diagnosis despite advancements in molecular diagnostics [37,38]. Initially, the procedure involved enclosing patients in a “plastic body bag” to facilitate sufficient sweat collection for sodium and chloride analysis, albeit with the attendant risk of hyperthermia [39]. In 1959, Gibson and Cooke introduced quantitative iontophoresis with pilocarpine, comprising four stages: local pilocarpine-induced sweat gland stimulation on the forearm, sweat collection, quantitative sweat electrolyte analysis (sodium, chloride), and result interpretation [6,12,40,41,42,43,44,45,46,47]. This test is deemed safe for diagnosing 98% of patients.

In 1986, the Macroduct system was introduced for sweat chloride concentration determination [48]. Subsequently, the Nanoduct system emerged, presenting a novel, expeditious, and straightforward diagnostic approach enabling sweat collection and conductivity analysis, measuring sodium chloride levels. Comparative assessments with the Macroduct revealed equivalent sensitivity (98% vs. 99%), albeit lower specificity (79% vs. 93%) and higher success rates with the Nanoduct system, as cited by Vernooij-van Langen et al. (2015) and Rueegg et al. (2019) [48,49]. Naerlich et al. (2020) posit that measuring conductivity using the Nanoduct system may aid diagnosis, albeit with lower specificity compared to chlorine measurement, thereby not being recommended for diagnostic purposes [46]. Current guidelines advise against employing conductivity as a confirmatory test, notwithstanding studies suggesting its potential as a definitive diagnostic tool [12,47,50]. Treggiari et al. (2021), as cited by Ren et al. (2021), advocate for sweat droplet number measurement as a more sensitive and specific diagnostic tool compared to the sweat test, although further research is warranted to establish its diagnostic utility [51,52].

B. Criteria for Sweat Test Utilization

**Figure 2:**
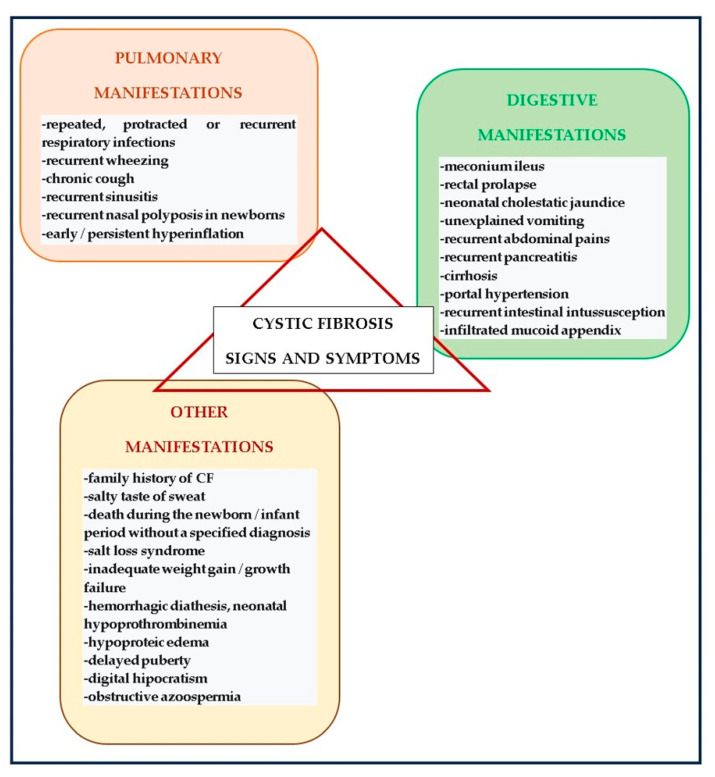
Signs and symptoms requiring a sweat test.

The indications prompting the employment of the sweat test encompass the following:

- Positive screening outcomes for CF.
- Clinical manifestations suggestive of CF, including the following:
  - In infants: meconium ileus, recurrent respiratory infections, steatorrhea, and failure to thrive;
  - In older children: chronic sinus-pulmonary infections;
  - In adults: azoospermia (Figure 2).
- Family history indicative of CF or carrier status [12,53,54,55].

C. Requirements for Conducting the Sweat Test

Executing the test necessitates adherence to specific conditions:

- The minimum gestational age must be 36 weeks, with testing ideally performed between 48 and 72 h postpartum to mitigate transiently elevated values observed within the initial 24 h. Testing beyond 4 to 6 weeks of age is optimal.
- Minimum weight at the time of testing should be 3 kg.
- The chosen testing site (typically the volar aspect of the forearm, occasionally the upper arm, thigh, or leg) must be devoid of inflammation, rash, or wounds to prevent contamination with other fluids or blood.
- Hydration with a minimum of 120 mL/kg body weight/day in the 24 h preceding the test is essential.
- A minimum sweat collection amount of 75 mg or 15 μL or 1 g/m^2^/minute is required to avert false positive or false negative outcomes attributable to insufficient sweat rates. In cases utilizing the Nanoduct system, 3 μL of sweat suffices, offering an advantage, particularly in neonates.
- Test duration should range from a minimum of 20 min to a maximum of 30 min.
- Application of a current with voltage below 15 V and intensity of 0.5 mA, gradually escalating to a maximum of 4 mA over a maximum duration of 5 min.
- Use of pilocarpine discs at concentrations of 2–5 g/L, a cholinergic agent stimulating the muscarinic receptors of sweat glands to activate secretion.
- Repetition after a minimum interval of one week and a maximum of two weeks to assuage parental concerns. Certain guidelines also allow for a single repeat test on the same day, albeit on the contralateral forearm [11,29,42,44,45,48,53,55,56,57,58,59,60,61,62].

D. Advantages, Limitations, and Challenges in Conducting the Sweat Test

The primary advantage of the sweat test lies in its capability to yield results on the same day. Conversely, a notable disadvantage may arise from the potential for ambiguous results that fail to accurately reflect disease progression and the number of quantity not sufficient (QNS) results [23].

Numerous challenges may be encountered during the execution of the sweat test:

- Inadequate sweat volume;
- Challenges in immobilizing pediatric patients;
- Difficulties in inducing sweating, particularly in infants;
- Incidence of skin burns, hives, irritation, and redness, accompanied by discomfort when electric current density surpasses 0.5 mA/cm^2^;
- Risk of electric shock and skin damage if the electrode metal makes direct contact with the skin;
- Potential issues related to contamination, evaporation, and inadequate collection duration [63].

Contraindications to performing the sweat test include corticosteroid treatment, presence of edema and dehydration, and open system oxygen therapy [53,55,56]. However, patients undergoing Flucloxacillin treatment may undergo the sweat test [56].

Various factors influence sweating rates, often associated with insufficient sweat production, including the following:

- Prematurity (2.4 times higher risk);
- Gestational age under 39 weeks (7.4 times higher risk);
- Low birth weight;
- Age and sex;
- African-American race;
- Skin condition and hydration status;
- Collection methodology;
- Testing conducted on different days;
- Environmental factors such as climate and family diet;
- Additional residual factors, including genetic modifiers that manifest as polymorphisms affecting responses to infections and inflammation [62,64,65].

Boys typically exhibit fewer active sweat glands with a higher sweat/gland rate and heightened responsiveness to cholinergic and beta-adrenergic stimulation compared to girls. Notably, dietary salt intake does not significantly alter electrolyte concentrations in sweat [11,44,66,67]. Additionally, colder weather during the winter months increases the risk of inadequate sweat production due to vasoconstriction, unlike warmer seasons [58]. Infants, particularly those who are malnourished or dehydrated, may encounter challenges in providing the requisite sweat specimen compared to older children [31,68].

The occurrence of samples exhibiting inadequate sweating rates stands at 5% among patients aged over 3 months. In cases of minimal sweat rate, there is a decrease in the concentration of electrolytes in sweat, thereby heightening the risk of sweat evaporation. The lower limit of electrolyte detection is set at 10 mmol/L, while the upper limit is capped at 160 mmol/L. Values exceeding 160 mmol/L are deemed physiologically implausible and necessitate retesting, as potential contamination or technical errors may have occurred [44,69]. Boys typically exhibit higher levels of chloride in sweat compared to girls, and individuals presenting with gastrointestinal symptoms demonstrate elevated chloride levels relative to those with respiratory symptoms [22]. According to the CF Foundation, the failure rate of tests is purportedly below 10%; however, in actuality, it ranges from 0 to 40% during the first 3 months of life [48].

Tests measuring chloride concentration in sweat, alongside sodium concentration contingent upon chlorine measurement and conductivity, are deemed suitable for CF diagnosis. Conversely, tests gauging osmolality are not endorsed for diagnostic purposes [53].

E. Interpretation of Sweat Test Results

The interpretation of sodium and chloride concentrations demands meticulous attention; as biological variations can significantly influence result interpretation [7]. Notably, the sweat test exhibits both intra- and inter-individual variability, even among patients sharing the same CFTR genotype. Traeger et al. (2014), observed a decline in chloride concentration during the first year of life, followed by an increase between 2 and 18 years, with a subsequent gradual decline after 18 years [70]. Conversely, Faria et al. (2016) noted an elevation in chloride concentration during the initial year of life, succeeded by a decline after the age of 2 years [67].

The interpretation of sweat test results varies across different guidelines and according to the Nanoduct manufacturer’s recommendations, as delineated in Table 1.

This change in values (equivocal range revised to 30–59 mmol/L from 40–59 mmol/L), coupled with genetic mutation analysis, enhances the likelihood of identifying individuals at risk of developing CF or those with CF [73]. The ECFS Standards of Care assert that there exists no international consensus on an age-specific lower limit [74]. The decision to set the minimum threshold at 30 mmol/L is substantiated by the detection of CFTR mutations even in individuals with values ranging between 30–40 mmol/L [75]. Notably, 10% of healthy adolescents may exhibit sweat test values exceeding 60 mmol/L. In such cases, administration of 9-α-fludrocortisone at a dosage of 3 mg/m^2^/day orally for 2 days enhances sodium reabsorption in normal sweat glands, resulting in normalization of electrolyte levels in healthy individuals [9].

Interpretation of the test results must also consider clinical symptoms, family history, age, and the fact that certain mutations may be associated with negative or equivocal outcomes, alongside instances of intermittent electrolyte elevations [12]. In cases where patients present with hyponatremia or hypochloremia, it is advisable to conduct the test following rehydration [69].

Test outcomes are contingent upon the proficiency of the personnel conducting the test and adherence to guidelines. Errors in sweat testing may arise due to protocol deviations, inadequate sweat collection, technical inaccuracies, or the misinterpretation of results. Alternative methods for stimulating sweating, such as sun exposure while covered with a blanket or inside a car, are not recommended due to the risks of burns, dehydration, and potential fatalities [76]. Moderate-intensity exercise (e.g., jogging in a climate-controlled environment) may serve as an alternative method for inducing sweating [77]. Diagnostic challenges can lead to delayed or incorrect diagnoses, treatment delays, and heightened anxiety among parents and healthcare providers [6].

False positive results may occur within the first 24 h of life or in patients with allergic conditions (e.g., atopic eczema, hypogammaglobulinemia), ectodermal dysplasia, glucose-6-phosphate dehydrogenase deficiency, endocrine disorders (e.g., untreated Addison’s disease, untreated hypothyroidism), metabolic disorders (e.g., certain glycogen storage diseases, mucopolysaccharidoses, malnutrition), urogenital tract disorders (e.g., Klinefelter syndrome, nephrosis). Treatment with Topiramate, an anticonvulsant, may elevate chloride values without clinical manifestations of CF, as it may induce oligohidrosis as an adverse reaction [78]. False negative results may occur in infants with hypoproteinemia-related edema, acute salt loss, or in individuals using mineralocorticoids [12,42,69,79,80]. False positive or false negative results may also stem from inadequate sweat collection, non-adherence to protocols, skin contamination, or lack of technical proficiency [53,81].

The conventional sweat test, which has been deemed the “gold standard” since the 1950s, remains the recommended diagnostic tool, even though approximately 10% of patients exhibit normal or equivocal values, a phenomenon often associated with specific mutations [82,83]. Individuals with an equivocal sweat test result should undergo monitoring for a minimum of 3 months within their first year of life and consider repeating the test between 9 and 12 months [24]. Clinical evaluation, along with various laboratory assessments (including fecal elastase, fecal fat analysis, liver function tests, sputum examination, chest X-ray, and screening tests to rule out other potential causes such as immunoglobulin E deficiency or α1-antitrypsin deficiency), as well as genetic testing, are imperative for these patients [9,84]. Moreover, the sweat test serves as a valuable biomarker for evaluating CFTR function in assessing the impact of in vivo modifying therapies such as modulators and potentiators [10,85,86].

In a 24-week, open-label, phase 3 trial, Zemanick et al. (2021) demonstrated the safety and effectiveness of Elexacaftor/Tezacaftor/Ivacaftor (ELX/TEZ/IVA) in children aged 6–11 years with at least one F508del-CFTR allele. On average, the sweat chloride levels decreased by 60.9 mmol/L, with a range between 58.2 and 63.7 mmol/L [87]. In a phase 3, single-arm, two-part, multicenter, multinational study (ARRIVAL) conducted by Rosenfeld et al. (2018), improvements were noted in sweat chloride levels. By week 2, sweat chloride decreased from a baseline mean of 104.1 mmol/L to 51.8 mmol/L, and by week 24, the mean sweat chloride concentration was 33.8 mmol/L [88]. Olivier et al. (2023) observed, in their study on CF patients receiving various CFTR modulators, that the mean sweat chloride concentration decreased by 59.3 mmol/L with ELX/TEZ/IVA and by 47.8 mmol/L in those transitioning from IVA/LUM or TEZ/IVA to ELX/TEZ/IVA [89]. Middleton et al. (2019), in a phase 3, randomized, double-blind, placebo-controlled trial on patients aged 12 years or older with cystic fibrosis and Phe508del-minimal function genotypes who received ELX/TEZ/IVA for 24 weeks, observed a decrease in sweat chloride concentration by 41.8 mmol/L [90].

#### 3.1.4. Sweat Conductivity

While the measurement of chloride concentration via pilocarpine iontophoresis is considered the most precise method for diagnosing CF, it does not directly reflect CFTR function [72]. Conductivity serves as a reliable means to distinguish between CF and non-CF individuals. As reported by Lezana et al. (2003), citing LeGrys et al., 45% of centers employ conductivity measurement—an easier technique—for CF diagnosis [91]. Sweat conductivity measurement exhibits commendable accuracy, offering heightened sensitivity and specificity. In this method, sweat conductivity tends to be approximately 15 mmol/L higher than sweat chloride due to the presence of unmeasured anions such as lactate and bicarbonate [7,72,81,92].

The interpretation of sweat conductivity values necessitates the consideration of age, as conductivity tends to increase with age, parallel to electrolyte concentration [36]. Moreover, the duration and cost of conductivity tests are comparatively lower than those for chloride determination in sweat [93].

#### 3.1.5. Genetic Mutation Analysis

Prior to the identification of the CF gene, CF diagnosis relied on clinical criteria and sweat tests. However, the discovery of the CFTR gene and advancements in laboratory techniques for mutation detection have significantly facilitated diagnosis [12]. Since 1989, the detection of genetic mutations has been feasible, with the CFTR gene located on chromosome 7q31.2, comprising approximately 250 kb of DNA with 27 exons separated by introns, and serving as a regulator of the amiloride-sensitive epithelial sodium channel (EnaC). The genes encoding the α, β, and γ subunits of ENaC have been identified [8].

CFTR encodes a transmembrane glycoprotein with 1480 amino acids and a molecular mass of 168 kDa, functioning as an electrolyte transporter at the apical membrane of epithelial cells. In certain tissues, CFTR plays a role in directly modulating or mediating bicarbonate secretion. Research by Choi et al. in 2001, demonstrated that CF patients with pancreatic insufficiency exhibit unmeasurable bicarbonate transport, whereas those without pancreatic sufficiency display reduced bicarbonate transport [94]. Tissues vary in their sensitivity to CFTR mutation, with some being more susceptible (e.g., different vessels) while others are less so (e.g., airway epithelium). In CF, sweat glands exhibit no histological abnormalities but demonstrate disruptions in sodium and chloride homeostasis due to CFTR dysfunction. A lack of CFTR functionality impedes sodium absorption and inhibits chloride reabsorption, leading to elevated concentrations of sodium and chloride in sweat, a sodium/chloride ratio exceeding 1, and disparities in transepithelial potential between extracellular fluids and sweat at the gland duct opening in CF compared to normal conditions [12].

The consequences of CFTR dysfunction often manifest before birth and may include embryological abnormalities such as the Wolffian structure, resulting in the bilateral congenital absence of the vas deferens—a cause of infertility [95]. It is estimated that maintaining 50% of normal CFTR levels is adequate for maintaining health. Consequently, carriers are not deemed at high risk for CFTR-related disorders [96].

A. Mutation Types and Their Association with Disease Severity

The identification of the CF gene has greatly enhanced the understanding of CF’s molecular mechanisms and introduced a novel diagnostic approach. Over 2000 mutations have been identified thus far, with approximately 83% linked to clinical CF, while around 200 are classified as polymorphisms, indicating their non-disease-causing nature, thus complicating molecular diagnostics [12,44,46,69,80,84,97,98].

According to the International Experts Update Guidelines, mutations are categorized into the following:

- CF-causing mutations;
- mutations with clinically variable consequences;
- non-CF-causing mutations;
- uncharacterized mutations [2,37,99].

CFTR mutations lead to ion transport defects characterized by deficient cyclic adenosine-monophosphate anion-dependent secretion and increased sodium-mediated absorption in the respiratory tract [100]. These variants exhibit varying effects on CFTR protein expression and function. Their absence or malfunction disrupts ion flow in the epithelial cells of affected organs in CF [2,37].

Genetic mutation analysis is crucial for confirming abnormal neonatal screening or sweat test results, identifying carriers, and conducting prenatal testing for carrier couples. However, this method can only detect a limited number of mutations [12,44,46,69,80,84,97,98]. Given that CFTR mutation frequency and distribution vary among populations, genetic testing should be tailored to each population’s variant frequency, thereby improving mutation detection rates [37,38]. In Romania, for instance, only 38 mutations can be detected. The F508del mutation is prevalent in 70% of European CF patients, with 49% being homozygous and 42% compound heterozygous [97]. Other mutations are rarer and depend on geographical and cultural factors [101].

Clinical severity is contingent on residual CFTR activity [65]. Genotyping has confirmed that certain genetic mutations are associated with mild phenotypes and normal or borderline sweat test electrolyte concentrations. Various mutation types have been identified, including missense, frameshift, splice site, nonsense, and deletions, with CFTR mutations classified into six groups based on their molecular and functional defects:

- class I: defective protein synthesis (e.g., G542X);
- class II: deficient protein processing, encompassing the most common mutation (F508 del—the initial mutation identified);
- class III: deficient regulation (e.g., G551D, S1255P);
- class IV: impaired function (e.g., 7117H, R334W, R347P);
- class V: reduced abundance (e.g., A455E);
- class VI: diminished protein stability (e.g., Q1412X).

Refs. [1,12,46,65,97,98,102,103]. Mutations may affect CFTR quantitatively, qualitatively, or both [69]. Class I, II, and III mutations are deemed to cause severe forms, whereas class IV, V, and VI mutations lead to milder forms due to functional CFTR protein. In the category of mild/variable mutations, mutations such as 3849+10 kb, D1152H, G85E, I1234V, R334W, and 5T are included, whereas severe mutations encompass F508del, W1282X, G542X, S549R, N1303K, Q359K/T360K, and 405+1G, among others [104]. The concept of “mild forms” and “severe forms” was introduced by Kerem et al. (1989) to elucidate CF’s clinical heterogeneity [82,105]. Patients with mutations in classes I–III typically exhibit pancreatic insufficiency, elevated lung function decline risk, and lower survival rates compared to those with mutations in classes IV–VI [106]. Childhood-onset suppurative lung disease and pancreatic insufficiency are common in patients with two mutations from classes I–III, whereas those with mild mutations (classes IV–VI) tend to develop pancreatic insufficiency later in life. However, the progression of patients with identical mutations, even within the same family, may differ, suggesting the influence of environmental factors (e.g., pollution, smoking, and pathogens) and secondary genetic factors altering CFTR function [12]. In 1950, the first instances of pancreatic insufficiency in patients with residual CFTR function due to mutations were documented [46]. Subsequently, in 1975, the first case of pancreatic insufficiency with a normal sweat test was reported [46,107]. Various genetic mutations, such as R117H, R334W, R347P, and P574H, are associated with pancreatic insufficiency, but there is no established correlation between elevated sweat test results or severe lung disease and genotyping [9]. For patients with the R117H mutation, it is crucial to distinguish between the presence of the 5T variant and the 7T variant. The coexistence of the 5T variant on the same chromosome results in a non-functional CFTR protein, leading to the onset of lung disease. Conversely, the presence of the 7T variant, characterized by a less severe splicing defect, is linked to milder lung disease or even the absence of symptoms [69,108].

US guidelines categorize patients as those with CF, those with CFTR-Related Disorders, and those without CF. On the other hand, EU guidelines classify patients into those with classic CF, those with CFTR dysfunction (non-classic/atypical forms), those with inconclusive forms, and those without CF [95].

The 5T allele located on intron 8 is a variant that exhibits variable penetrance, leading to an inefficient splicing of exon 9. While the 5T allele may manifest as the classical CF phenotype or a milder form, without evidence of CFTR dysfunction, it alone does not cause CF [24,95,108]. This allele is frequently observed in males with vas deferens absence, pancreatitis, atypical sino-pulmonary disease, or in newborns with hypertrypsinemia and normal sweat tests [109]. The 7T allele is associated with CFTR-Related Metabolic Syndrome, while the 9T and 11T alleles are rarely problematic [24].

B. Recommendations for Genetic Mutation Determination

Genetic testing is advised in different scenarios, including neonatal screening with a positive sweat test, negative sweat test with suggestive CF clinical signs, positive neonatal screening, the presence of CF clinical signs or family history, and equivocal sweat test results in two separate tests, when both parents are carriers, one or both parents have CF, or one parent is a carrier and the other is unavailable for testing, in husbands of carrier women for CF [29,110]. For patients exhibiting CF-specific symptoms, genetic testing followed by a sweat test is recommended when family mutations are known, only one mutation in the family is identified, or if sequencing fails to reveal two disease-causing mutations [110].

C. Limits and Disadvantages of Genetic Testing

While molecular analysis can confirm diagnosis, it comes with certain limitations:

- Large number of mutations (over 2000).
- Low cost-effectiveness ratio.
- Absence of common mutations in some populations [83]. A drawback is the time required to provide results, typically taking at least 1–2 weeks.

D. Interpretation of Genetic Testing

Patients harboring two CFTR mutations with known pathogenicity are diagnosed with CF. The identification of dual mutations not only confirms the diagnosis but also facilitates genetic counseling and provides insights into phenotypic variations, such as exocrine pancreas status [111]. For patients with 0–1 mutations, complementary sweat testing is necessary to ascertain whether they have CF or are carriers. Extended exon analysis is also recommended in such cases. It is important to note that the absence of two CF-causing CFTR mutations, despite clinical and laboratory signs, does not exclude a CF diagnosis [29,74].

Given that commonly used tests may not detect all CFTR gene mutations, a negative test does not always indicate a normal CFTR genotype. Genetic mutation analysis serves not only to identify carriers but also for prenatal diagnosis in high-risk pregnancies.

A small percentage of patients (1–2%) exhibit characteristic symptoms but have normal or equivocal sweat test results. In such cases, genetic testing becomes imperative. Mutations possibly associated with normal or equivocal values include R117H, D1152H, A455E, G551S, 2789+5G-A, and 3849+10 kb C˃T. Conversely, the S1455X mutation may lead to elevated ST values in the absence of other symptoms (normal pulmonary function, sputum examination, and exocrine pancreatic manifestations) [12,72].

#### 3.1.6. DNA Sequencing Analysis of CFTR Gene

In certain centers, it is possible to conduct exon sequencing along with specific intronic regions, enabling the detection of 98% of CFTR mutations [69]. CFTR gene sequencing aids in identifying mutations with unclear genotype–phenotype correlations, likely influenced by environmental factors [112]. Next-Generation Sequencing scans for entire candidate genes influencing clinical outcomes, allowing the detection of deletions, duplications, and over 97% of CFTR mutations associated with CF, thereby expanding the number of variants with unknown clinical significance. Complete genetic DNA sequencing is recommended for patients with a clinical diagnosis of CF but incomplete genotype [95,97]. However, despite sequencing, there are cases with CF-like clinical presentations but normal CFTR genotype due to unidentified mutations outside the sequenced areas [46,101]. However, in the context of numerous screening programs, the identification of carriers is often regarded as undesirable.

In cases of clinical CF signs with equivocal ST, alternative tests like nasal NPD, followed by DNA sequencing, are advised. If sequencing remains inconclusive, an examination of ion transport in gastrointestinal tissues (small intestine or rectal mucosa) is recommended [101].

Patients with monosymptomatic presentations (pancreatitis, absence of vas deferens, or bronchiectasis), where CFTR dysfunction does not meet CF criteria, are categorized as having CFTR-Related Disorders, conditions resembling CF symptoms [29]. These patients may have residual mutations, severe mutations shared with CF-affected relatives, other severe complex alleles with residual CFTR activity, or non-CF-causing mutations [113]. Given the diversity of CFTR mutations and related disorders, comprehensive molecular screening covering all 27 exons and regulatory regions (5′UTR, 3′UTR, and partially intronic regions) is necessary [97].

#### 3.1.7. Nasal Potential Difference (NPD)

To assess the functional implications of CFTR mutations and distinguish between CF-causing mutations and silent variants, an additional “gold standard” test is required [91]. NPD measurement offers insight into modified or altered ion transport across the respiratory epithelium of CF patients in vivo. Introduced by Mike Knowles in 1995, NPD measurement serves as a complementary tool to sweat testing and genetic mutation analysis. However, due to its technical complexity, NPD is not routinely performed in clinical practice and is primarily utilized in research settings [6,114].

In cases where the consequences of CFTR mutations are unclear, employing NPD as a diagnostic tool is recommended [114]. It is important to note that the presence of nasal inflammation (e.g., allergic rhinitis, viral infections) can potentially alter ion transport, leading to false-negative results. Other factors such as pollution, pulmonary exacerbation, and physiological variations in estrogen levels during menstruation can also influence NPD variability [115].

NPD assesses relative ionic conductance by measuring transepithelial voltage changes in surface epithelial cells of the intranasal mucosa in vivo. The loss of CFTR-dependent anion conductance results in a characteristic hyperpolarization pattern, which is associated with specific transepithelial voltage changes in response to transport inhibitors and agonists [116,117].

#### 3.1.8. Intestinal Current Measurement (ICM)

ICM emerges as a novel ex vivo technique developed to assist in diagnosing CF in patients exhibiting mild or sub-clinical symptoms, as well as those with ambiguous or borderline ST results, or rare CFTR mutations. This method offers several advantages, including the following:

- Easy accessibility to intestinal tissue at any age.
- Minimal to no tissue damage or remodeling due to bacterial or viral infections.
- Potential for testing novel CFTR therapeutics in human epithelium ex vivo without risking patient safety.
- Capability to detect very low levels of functionally active CFTR.
- The feasibility of performing the test without sedation across all age groups.
- Painless procedure completed in under 5 min [118].

#### 3.1.9. New Non-Invasive Diagnostic Methods

Despite ongoing efforts to enhance iontophoresis quality, inadequate sweat sample collection remains a prevalent issue in CF diagnosis [11]. Novel diagnostic approaches and techniques employ alternative analytical methods and investigate factors to minimize sweat volume needed for accurate sweat chloride measurement [49,119]. However, the high failure rates and limited performance in clinical trials hinder the widespread adoption of these methods for the large-scale screening of suspected cases [48]. Additionally, the need for laboratory-scale analytical instruments and controlled environments for assessment further restricts their utility in remote settings [120,121,122].

Conventional sweat collection methods involving skin-mounted straps, as well as iontophoresis itself, can be challenging, particularly for infants and newborns with delicate skin who often produce insufficient sweat. Hence, there is an urgent need for a straightforward and rapid sweat collection and chloride analysis system, particularly in age groups where iontophoresis is particularly challenging, such as full-term and especially premature newborns and small infants.

New generations of bio-integrated sensors, a form of wearable technology, offer potential solutions to overcome ST limitations. These sensors provide non-invasive and close integration with various body surfaces [123]. Emaminejad et al. (2017) demonstrated the feasibility of a portable electrochemical sensor capable of locally stimulating sweat and simultaneously analyzing its chloride content via a single battery-powered platform, paving the way for real-time, in situ sweat chloride measurement [124]. Other studies explore commercial iontophoresis systems with instrumented collection platforms to minimize sweat volume requirements through alternative quantification methods such as conductivity, potentiometry, fluorimetry, and Ultrafast Nonlinear Imaging and Spectroscopy [93,125,126,127,128].

While these approaches offer potential in reducing sweat collection needs, they rely on intricate analytical methods currently not endorsed by CF diagnosis clinical guidelines [29]. Overcoming challenges such as lengthy sensor stabilization times during calibration, individual sensor variability, and the development of intricate battery-powered electronic circuits are fundamental hurdles in their widespread use [120]. Recently developed techniques propose a solution to these limitations by employing devices utilizing arrays of microfluidic channels integrated into soft, elastomeric substrates for passive sweat capture, storage, and quantitative analysis [129,130,131]. Demonstrations of these epidermal microfluidic devices (“epifluidic” devices) show how they utilize natural pressure generated by sweat glands to guide sweat flow, along with colorimetric reagents and wireless methods, such as integrated sensors, for real-time sweat biomarker analysis in diverse biochemical environments without evaporation risks [132,133,134,135,136,137,138].

To develop a simple, non-invasive CF test, Ray et al. (2021) introduced an adhesive microfluidic device (referred to as a “sweat sticker”) for real-time sweat capture and analysis using colorimetric readouts. Testing and validating this device in CF patients demonstrated its ability to monitor sweat chloride concentrations using smartphone images of sweat stickers adhered to the skin, showing promising results. The device’s intimate skin coupling exhibits nearly perfect efficiency in sweat collection without leakage. Clinical validation studies in CF patients and healthy subjects across various age groups support its clinical equivalence to existing devices in terms of determination accuracy and show significant reductions in errors due to sweat leakage. Portable microfluidic technologies and smartphone-based assays represent a significant advancement in accurate CF diagnosis [123].

### 3.2. Diagnosing CF

In many instances, clinical symptoms are distinctive enough, with elevated electrolyte levels being indicative, thus rendering genetic analysis unnecessary. Nevertheless, mutation analysis can be beneficial in affirming the diagnosis, identifying carriers, and facilitating prenatal diagnosis [57].

As per guidelines from the CF Foundation (USA) and the European Cystic Fibrosis Society (ECFS), ST remains the primary diagnostic tool for CF [69]. Often, CFTR mutation analysis is the initial or sometimes the sole test conducted, although it is advisable to perform ST before genetic testing. Ruling out CF solely based on the absence of two CFTR mutations can result in missed diagnoses [139,140].

Diagnostic criteria for CF encompass the following:

- The presence of clinical symptoms;
- A family history of CF;
- The identification of two CF gene alleles indicating dysfunctional CFTR;
- Elevated electrolyte concentration in ST;
- Increased Nasal Potential Difference [83,91].

In the absence of a characteristic phenotype, the recommendation is to identify two disease-causing mutations or conduct ST with values exceeding 60 mmol/L or showing abnormal NPD [69].

As per CF Foundation criteria (2015), individuals with positive neonatal screening plus two mutations or suggestive CF signs or meconium ileus should confirm the presumptive diagnosis with ST. For patients not benefiting from neonatal screening with equivocal ST, investigating CFTR function (NPD or intestinal current measurement) is advised, leading to classification as CFTR-Related Metabolic Syndrome (CRMS) if the genetic test remains inconclusive [29].

According to the Australasian Guideline (2017), positive neonatal screening with two CFTR mutations warrants ST confirmation. With only one mutation, ST is also recommended to differentiate from probable carriers. If clinical signs specific to CF are present, ST should be performed within five days of a positive neonatal screening result [80]. Similar recommendations are outlined in the National Institute for Health and Care Excellence (NICE) guideline [110].

According to American Diagnostic Criteria, CF diagnosis relies on identifying two CFTR mutations plus elevated chlorine levels exceeding 60 mmol/L in ST [91].

Per the ECFS Standards of Care (2014), clinical signs suggestive of CF alongside equivocal ST and 0–1 mutations necessitate follow-up in a CF clinic, with additional tests conducted and complications monitored. Patients with suggestive signs and equivocal ST should undergo sequencing and NPD determination. Positive results indicate CFTR dysfunction, classifying the patient as having atypical CF (non-classical) [141].

As per the European Diagnostic Working Group, patients are categorized as having classic CF if they exhibit one or more phenotypic characteristics, ST above 60 mmol/L, pancreatic insufficiency/sufficiency, and a severe prognosis. Patients without these criteria fall into the non-classic or atypical category, many of whom have mild lung disease and pancreatic insufficiency [142].

The Royal College of Paediatrics and Child Health suggests that while the presence of two CFTR gene mutations can confirm the diagnosis, demonstrating two mutations is not imperative. CFTR mutation confirmation, coupled with clinical symptoms, does not necessitate ST confirmation [143]. However, in England’s NBS program, ST is recommended after positive NBS if two CFTR mutations are present [110] (Table 2).

The molecular diagnosis of CF poses challenges due to the increasing diversity of variants and genotypes, along with the complexities involved in assessing their impact [2]. Clinical variability arises from the varied molecular effects of different mutations affecting the CFTR gene. Additionally, the phenotype may be modulated by other genes and exposure to diverse environmental factors [144].

Diagnosing the classic form of CF relies on clinical symptoms indicative of CFTR dysfunction (elevated sweat chloride concentrations, decreased nasal potential difference), followed by the confirmation of the presence of two mutations. Distinguishing between CF and CFTR-Related Disease necessitates thorough clinical assessment [145]. While the combination of ST and genetic analysis simplifies diagnosis in many cases, some patients fail to meet all diagnostic criteria, particularly those with organ-specific damage [146].

CF diagnosis may also occur in asymptomatic individuals with positive test results (newborn screening, sweat test, and confirmed genetic testing) [110].

### 3.3. Phenotype–Genotype Correlations

Phenotype–genotype correlations in CF are intricate, involving the interplay of CFTR gene mutations, genetic modifiers, chloride transport, interaction with other ion channels, intracellular CFTR function, the tissue expression of CFTR, and tissue response to CFTR mutation alongside exposure to various environmental factors. There are instances where clinical manifestations, CFTR genotype, and electrolyte measurements present conflicting evidence regarding CF diagnosis. Some cases exhibit symptoms suggestive of CF, yet ST yield normal or equivocal results, with patients displaying pancreatic insufficiency, improved nutritional status, and older age at diagnosis. Factors beyond CFTR dysfunction may contribute to a non-classical phenotype [12]. While most diagnoses occur during childhood, some cases go undetected or are only diagnosed in adulthood [7].

The clinical phenotype varies among patients depending on residual CFTR function [106]. The identification of numerous CFTR gene variants and diverse molecular mechanisms responsible for CF contributes to phenotypic diversity [2,101]. Even patients sharing the same CFTR genotype may exhibit differing phenotypes [101,147]. Variations in sweat chloride values have been observed across different CFTR mutation classes, and patients with identical CFTR genotypes may display sweat test variations [61]. Environmental factors, such as *Burkholderia cepacia* infection, can influence disease phenotype [90]. Clinical phenotype variability reflects genetic variation in the genome [148]. CFTR defects give rise to organ-specific clinical phenotypes unique to each affected organ [149].

Certain mutations, such as F508del, G542X, G551D, N1303K, W1282X, R553X, 621+1G˃T, 1717-1G˃A, and R1162, are associated with the classic phenotype, while R117H is linked to the non-classical phenotype [69].

Severe phenotypes are characterized by elevated sweat chloride values, early-onset pancreatic insufficiency, and severe lung disease, while mild phenotypes exhibit lower sweat chloride values, pancreatic insufficiency, variable lung disease severity, and no history of meconium ileus [150].

Genotype plays a crucial role in lung phenotype and survival [151]. CFTR genotype dictates the extent of pancreatic exocrine dysfunction and correlates with abnormal sweat chloride values and male reproductive malformations. Disease severity has been observed to correlate with genotype [152]. The relationship between sweat chloride values and mortality varies based on genotype, with the highest mortality observed in patients with the R117H/F508 del mutation on a background of 5T [106].

The impact of CFTR genotype on clinical phenotype varies among organs, with the vas deferens being the most sensitive and the lungs being the least affected by reduced CFTR function [37]. Achieving phenotype–genotype correlations requires combining epidemiological data with in vitro or in vivo functional analysis and comprehensive clinical information [97]. Due to the extensive mutation spectrum and considerable clinical variability, establishing genotype–phenotype correlations, except for the most common mutations, is challenging [82].

Disease severity correlates with organ sensitivity to CFTR dysfunction and the level of functional protein influenced by mutation type [153]. Kiesewetter et al. (1993) observed that the R117H missense mutation, where arginine is replaced by histidine at position 117 of the CFTR protein, affects splicing efficiency, suggesting that the mutation’s context plays a significant role in disease manifestation [101,154]. R117H is associated with a mild disease phenotype and may coexist with another variant on the same allele leading to a classic or severe phenotype. When paired with the 7T variant, the phenotype may be normal. The 5T variant, when coexisting with the R117H mutation and a second disease-causing mutation, leads to lung disease. However, patients with the 7T variant alongside the R117H mutation and a second disease-causing mutation either do not develop lung disease or exhibit mild lung symptoms, as I mentioned before [69,108]. Phenotype–genotype correlation should not be solely relied upon for CF patient prognosis [37].

#### 3.3.1. Relationship between Phenotype, Genotype, and Pancreatic Function

The impact of CFTR mutations is closely linked to the pancreatic phenotype and the quantitative assessment of exocrine pancreatic function. Research by Ahmed et al. (2003) emphasized that individuals homozygous or compound heterozygous for class I, II, and/or III CF mutations typically experience severe pancreatic involvement, often leading to pancreatic insufficiency or progressive development thereof. Conversely, patients with class IV mutations tend to have milder pancreatic disease [155]. Notably, 18% of patients with idiopathic pancreatitis have been found to harbor disease-causing mutations, suggesting a potential association between idiopathic pancreatitis and CFTR mutations [156].

Castellani et al. (2008) noted that individuals with splicing mutations (e.g., 621+1G>T, 1525-1G>A, 711+1G>T) exhibit a variable phenotype ranging from mild lung disease and pancreatic insufficiency to severe multi-organ involvement [37]. The degree of correlation between CFTR genotype and phenotype varies, with stronger correlations observed for pancreatic function status compared to lung disease severity [4,97,157].

There exists a discernible association between genotype and pancreatic status, where class I–III mutations are typically linked with pancreatic insufficiency, while those in classes IV–V are associated with milder pancreatic involvement, although certain mutations may exhibit overlapping effects on pancreatic function [37]. Moreover, CF patients with pancreatic insufficiency and severe mutations are at an elevated risk of progressing to pancreatic insufficiency over time [158]. The development of cystic fibrosis-related diabetes is contingent upon the residual function of CFTR.

Pancreatitis can manifest as a symptom of CF or CFTR-Related Disease [159]. The genotype–phenotype correlation in pancreatitis differs from that observed in other organ manifestations, with genotypes exhibiting mild phenotypic effects posing a higher risk for pancreatitis compared to those with moderate or severe effects [3]. Pancreatic insufficiency is more prevalent in patients with 1–2 mild mutations, while homozygotes or individuals with two severe mutations are more likely to develop pancreatic insufficiency [4]. Certain aspects of the CF phenotype are determined by the specific CFTR mutation, while others are influenced by additional factors [8].

Studies, such as the one conducted by Mark et al. (1999), have shown that patients with pancreatic insufficiency frequently harbor class I–III mutations (e.g., F508del, W1282X, N1303K, S549R, 1677delTA, R117L, 4016insT, G544S, 2423delG, V754M, and 741T→G), while those with pancreatic sufficiency often present with class IV–V mutations (e.g., R117H, A455E, R334W, R347P, L206W, and P67L) [69,160]. Notably, the L997F mutation is frequently associated with pancreatitis [157].

#### 3.3.2. Relationship between Phenotype, Genotype, and Pulmonary Function

The interplay between genotype and pulmonary phenotype in CF patients is intricate, with individuals harboring class I–III mutations typically experiencing a more rapid decline in lung function [149]. Virtually all CF patients develop rhinosinusitis characterized by viscous mucus, impaired muco-ciliary clearance, and inflammation in the nasal and sinus cavities, leading to symptoms such as anosmia, headache, facial pain, and nasal congestion [161]. While Southern et al. (2004) suggest an increased prevalence of CFTR mutations in patients with isolated rhinosinusitis or bronchopulmonary aspergillosis, Noone et al. (2001) argue that these conditions may be less commonly associated with CFTR mutations and could be influenced by non-CFTR genetic changes and environmental factors [101,162].

Studies, including one by Highsmith et al. (1994) have identified mutations such as 3849+10 kb C˃T in intron 19 of the CFTR gene in patients with severe lung disease but normal sweat chloride test (ST) results, indicating a lack of correlation between ST and lung function in these cases [163]. Similarly, mutations like 3849+10 kb C>T, I119V, R334W, and P67L have been associated with equivocal ST values [101,164]. However, certain mutations, such as the missense mutation S1455X, can lead to abnormal ST values without being associated with CF [101]. The absence of a clear correlation between genotype and lung disease severity may be attributed to factors such as the young age of patients in studies, limited disease progression, and the inclusion of less common mutations in adults [151].

Environmental factors and genetic modifiers likely play a significant role in modulating the severity of lung disease in CF patients, as suggested by the weak correlation between CFTR genotype and lung phenotype [148,165,166,167]. The complexity of framing the pulmonary phenotype arises from variations in disease onset, progression, and severity due to intrinsic and extrinsic factors [168]. Although chronic rhinosinusitis is common in CF, the impact of the F508del mutation on its severity remains inconclusive, as indicated by Abuzeid et al. (2018) in a retrospective study [169].

#### 3.3.3. Liver Damage in the Context of Phenotype–Genotype Relationship

Certain CFTR mutations, such as R248/[H939;H949L], have been associated with hepatopathy characterized by elevated transaminase levels, typically observed in CF patients with good nutritional status and pancreatic insufficiency [170]. In a multicenter study referenced by Salvatore et al. (2002), mutations in α1-antitrypsin and mannose-binding lectin were identified as independent risk factors for liver disease in CF patients. Patients carrying the 1259insA/[H939;H949L] mutation may present with meconium ileus, while those with the F508del mutation often exhibit severe pulmonary manifestations [171].

A prospective study by Colombo et al. (2002) found the F508del mutation in approximately 51–55% of CF patients with cystic fibrosis liver disease (CFLD), suggesting a correlation between specific CFTR genotypes and variable liver phenotypes [172]. The liver phenotype in CF patients is influenced by environmental factors, medications, chronic infections, and malnutrition, which may affect the progression of CFLD [173,174].

#### 3.3.4. Relationship between Phenotype, Genotype, and Reproductive Function

In males, CFTR plays a crucial role in regulating expression in the testes and epididymis, impacting sperm maturation. Reduced or absent CFTR activity leads to increased viscosity of epididymal fluid and male infertility [175]. Studies on CFTR gene mutations have shown that infertile men with obstructive azoospermia resulting from the congenital absence of the vas deferens or epididymal obstruction have a higher prevalence of CFTR gene mutations, including D979A, R258G, and M952T [160]. Mutations and polymorphisms in the CFTR gene, such as (TG)m and Tn polymorphic loci in intron 8 at the splice acceptor site of exon 9, can also contribute to male infertility [176]. A congenital absence of the vas deferens is often associated with two mutations, typically comprising a severe mutation paired with a mild one or two mild mutations, compared to individuals with idiopathic epididymal obstruction [160,175]. The presence of the 5T variant is linked to the bilateral congenital absence of the vas deferens phenotype, although some patients may not have mutations on both gene copies due to molecular heterogeneity and the diverse spectrum of CFTR mutations [176].

In CF patients, the variability in phenotype among individuals with the same genotype or homozygosity for non-sense mutations suggests the involvement of environmental and/or genetic factors. Discordant phenotypes observed in CF-affected relatives imply the influence of genes other than CFTR on CF phenotypes [171]. Genetic modifiers affecting meconium ileus have been identified on chromosome 19q13.2 [167]. Brown et al. (2006) suggest that typical and atypical lung phenotypes can be distinguished after the age of six, marking the youngest age for distinguishing patients with pancreatic sufficiency from those with insufficiency [168].

Decaestecker et al. (2004) found no significant differences in age at diagnosis, ST values, weight, or complications between patients with the G85E mutation compared to those with F508del/F508del mutations [150]. Similarly, the V456A mutation, as studied by Ruwan et al. (2019), has been associated with equivocal ST values and mild to moderate forms of the disease, potentially leading to delayed diagnosis [38]. Moussafi et al. (2006) investigated the D1152H class IV mutation in CF patients aged 8 months to 56 years and noted its association with a broad clinical spectrum, providing valuable information for genetic counseling [177]. Sosnay et al. (2013) analyzed genotype–phenotype correlations in 39,696 CF patients and identified 159 variants meeting both clinical and functional diagnostic criteria [178].

A small subset of CF patients has mutations associated with normal ST [100]. Approximately 2% of CF patients exhibit an “atypical” phenotype characterized by symptom onset in adolescence or adulthood, suggestive symptoms, pancreatic insufficiency, and ST below 60 mmol/L [109,153]. In 1997, Ho et al. suggested that the weak correlation between clinical examination and genotype in CF could be attributed to chlorine secretion having a greater influence on clinical status than genetic mutations [147].

## 4. Discussion

While significant progress has been made in diagnosing CF, there are still gaps in our understanding and approaches to diagnosis. However, these approaches may not capture all cases of CF, especially those caused by rare or novel mutations. There is a need for additional biomarkers that can facilitate early detection, particularly in cases where genetic testing is inconclusive or unavailable.

CF can present with atypical symptoms or in conjunction with other conditions, leading to diagnostic challenges. For example, individuals with CF may have normal sweat chloride levels or present with primarily respiratory or gastrointestinal symptoms. More research is needed to better characterize these atypical presentations and develop diagnostic algorithms tailored to different patient populations. Access to specialized diagnostic tools, such as the sweat test and genetic testing, may be limited in certain regions or healthcare settings. This can result in delays or disparities in diagnosis, particularly in underserved populations. Addressing these disparities requires efforts to improve access to diagnostic testing and expertise in CF diagnosis.

While newborn screening has greatly improved the early detection of CF, it can also lead to challenges in interpretation, particularly in cases of inconclusive or borderline results. There is a need for standardized protocols and guidelines for the follow-up testing and clinical management of infants with positive or inconclusive newborn screening results.

While there is growing interest in integrating multi-omics data (e.g., genomics, transcriptomics, and proteomics) for CF diagnosis, there are challenges in analyzing and interpreting these complex datasets. Methods for integrating and harmonizing multi-omics data from diverse sources are needed to realize the full potential of omics-based diagnostics in CF. While biomarkers for CF diagnosis exist, there is a lack of reliable biomarkers for predicting disease progression and monitoring treatment response. Identifying biomarkers associated with disease progression and treatment outcomes could improve the personalized management of CF and guide therapeutic decisions.

Current diagnostic tests for CF often require specialized equipment and laboratory facilities, leading to delays in diagnosis and limited access in resource-limited settings. There is a need for the development and validation of point-of-care diagnostic tests that are rapid, affordable, and suitable for use in diverse healthcare settings. While CF is primarily a genetic disorder, environmental and epigenetic factors may also influence disease expression and progression. Further research is needed to elucidate the role of environmental exposures, epigenetic modifications, and gene–environment interactions in CF pathogenesis and diagnosis.

Addressing these gaps will require collaborative efforts across disciplines, including genetics, molecular biology, epidemiology, and clinical medicine, to improve the early detection, accuracy, and personalized management of CF.

The extension of lifespan in CF patients is linked with the presence of numerous accompanying conditions, with 32% of adults aged 20–64 having over five comorbidities [179]. A timely diagnosis of CF enhances the likelihood of achieving favorable therapeutic outcomes and postponing the onset of accompanying conditions. The implementation of neonatal screening for CF diagnosis represents a significant advancement. Positive cases are subjected to additional tests, with genetic testing playing a crucial role in determining case management. Genetic tests predominantly cater to the Caucasian population, often yielding false negatives in non-Caucasian populations [180].

A considerable challenge lies in classifying uncertain or ambiguous cases, as it is imperative to monitor patients with positive screenings lacking clear diagnostic indicators. Currently, chloride concentration measurement via pilocarpine iontophoresis remains the most accurate method. Assessing the functional implications of CFTR mutation and distinguishing between disease-causing mutations and silent variants is achieved through NPD measurement. Investigating rare CF mutations is vital, as they can provide valuable insights into disease prognosis and genotype–phenotype correlations, thus enhancing patient care and outcomes. Due to the diverse clinical phenotypic variations among CF patients, diagnosis can be intricate. The time taken for diagnosis, alongside factors like lung disease severity, nutritional status, and treatment options, can significantly impact prognosis. Recognizing the advantages of early diagnosis is crucial for healthcare providers and families.

Several biomarkers have been developed and utilized for the diagnosis of CF. Elevated levels of chloride in sweat samples are a hallmark diagnostic feature of CF. Sweat chloride testing, typically performed using quantitative pilocarpine iontophoresis, is a primary diagnostic tool for CF. Mutations in the CFTR gene are the underlying cause of CF. Testing for specific CFTR mutations, such as ΔF508, can aid in confirming the diagnosis of CF, especially in cases with inconclusive sweat chloride results. NPD testing measures the difference in electrical potential between the nasal epithelium and a reference electrode. Abnormalities in NPD, reflecting altered ion transport across epithelial cells, are observed in individuals with CF and can contribute to the diagnostic evaluation. Various inflammatory markers, such as interleukins (e.g., IL-8), tumor necrosis factor-alpha (TNF-α), and C-reactive protein (CRP), may be elevated in individuals with CF. While not specific to CF, these biomarkers can provide additional diagnostic and prognostic information, particularly regarding disease severity and exacerbations. Imaging modalities such as chest X-rays, computed tomography (CT) scans, and magnetic resonance imaging (MRI) can reveal characteristic features of CF lung disease, including bronchiectasis, mucous plugging, and airway wall thickening. These imaging biomarkers aid in the diagnosis and monitoring of CF-related lung pathology. Identification of specific bacterial pathogens in respiratory samples, such as *Pseudomonas aeruginosa* and *Staphylococcus aureus*, is integral to CF diagnosis and management. Microbiological cultures and molecular assays help detect and monitor microbial colonization in CF patients.

These biomarkers, used alone or in combination, play a crucial role in diagnosing CF, monitoring disease progression, guiding treatment decisions, and assessing therapeutic efficacy in individuals with CF. Even if several key biomarkers have been intensively analyzed, studies in this direction should continue in order to discover more sensitive, efficient and easily accessible methods for all patients. Ongoing research continues to explore novel biomarkers and diagnostic approaches to improve CF diagnosis and management.

The introduction of novel therapies in CF has revolutionized the landscape of this disease, shifting it from a childhood fatality to a condition offering survival prospects. However, current therapies are tailored only to specific gene mutations [181]. The presence of a multidisciplinary clinical team plays a pivotal role in translating existing evidence into practice, ensuring standardized care, with the active involvement of CF patients in defining their care [182]. Diagnosis confirmation and treatment initiation are just initial steps in CF patient management. Alongside specific treatment, it is imperative to conduct screenings to detect anxiety and depression in both patients and their caregiving family members. The clinical assessment of diagnosed cases of anxiety or depression and the implementation of pharmacological treatment along with psychological interventions are essential, as anxiety and depression can detrimentally affect therapeutic adherence.

Future CF clinical trials should focus on identifying novel diagnostic methods with enhanced specificity and sensitivity.

## 5. Conclusions

Given the gravity of the condition, it is imperative to diagnose CF as early as feasible to promptly initiate the intricate treatment regimen. Despite the accessibility and advancements in genetic testing, sweat chloride testing (ST) remains the current gold standard in CF diagnosis, serving as a reliable measure of CFTR function. Nonetheless, genetic testing should be integrated into routine assessments to ascertain CF diagnosis or carrier status. Instances where diagnosis remains ambiguous post-ST and genetic mutation analysis necessitate confirmation through additional CFTR function tests, although some may pose challenges in infants. The CF phenotype is influenced by genotype, with genotype–phenotype correlations proving crucial for genetic counseling, particularly in cases involving two carriers without an affected child but who have undergone screening.

## 6. Future Directions

Future investigations into diagnosing CF are poised to concentrate on several pivotal domains aimed at enhancing early detection, precision, and tailored treatment.

- Sustained exploration of CF’s genetic and molecular underpinnings remains crucial for pinpointing novel mutations, deciphering genotype–phenotype correlations, and unveiling fresh biomarkers linked to CF pathophysiology. This encompasses delving into the involvement of non-CFTR genetic modifiers and epigenetic elements in disease manifestation and progression.
- Progressions in functional assays to gauge CFTR functionality and the identification of innovative biomarkers stand as linchpins for augmenting diagnostic precision and monitoring disease evolution. Prospective inquiries might center on crafting more refined biomarker arrays, reflecting diverse facets of CF pathophysiology like inflammation, infection, and organ malfunction.
- Research endeavors could persist in honing newborn screening methodologies and algorithms to enhance the sensitivity, specificity, and cost-effectiveness of CF screening initiatives. This might entail assessing the efficacy of emerging screening technologies, fine-tuning screening thresholds, and probing the feasibility of expanded screening protocols covering additional CF-linked conditions.
- The development and validation of point-of-care diagnostic apparatuses for CF-associated biomarkers represent a focal point for forthcoming research. These compact and swift testing platforms hold promise for streamlining diagnostic workflows, enabling early detection in remote or resource-constrained settings, and facilitating personalized treatment decisions at the bedside.
- The amalgamation of cutting-edge imaging modalities with artificial intelligence algorithms holds potential for refining the precision and efficacy of CF diagnosis and monitoring. Prospective investigations could explore the utility of AI-driven image analysis for quantifying lung involvement, the early identification of structural alterations, and forecasting disease trajectory.
- An integrative scrutiny of multi-omics datasets, encompassing genomics, transcriptomics, proteomics, metabolomics, and microbiomics, is indispensable for unraveling the intricate molecular mechanisms underlying CF and identifying fresh diagnostic and therapeutic targets. Advanced bioinformatics methodologies and machine learning algorithms are poised to play pivotal roles in deciphering expansive omics datasets and extracting actionable insights.
- Subsequent studies may concentrate on implementing personalized medicine paradigms in CF diagnosis and treatment. This entails stratifying patients into molecularly defined subgroups predicated on their genetic and molecular profiles, prognosticating individual treatment responses, and customizing therapeutic interventions to target precise disease mechanisms.
- Longitudinal cohort investigations and analyses of real-world data are primed to furnish invaluable insights into the natural progression of CF, the trajectories of disease evolution, and the ramifications of interventions on clinical outcomes. These endeavors will aid in fine-tuning diagnostic criteria, optimizing treatment algorithms, and shaping clinical practice guidelines.

Collectively, future inquiries into diagnosing cystic fibrosis are positioned to adopt a multidisciplinary and translational approach, integrating advancements in genetics, molecular biology, imaging, data science, and clinical research to ameliorate the early detection, precision, and tailored management of CF patients.

## Figures and Tables

**Table 1:** Different interpretations of the sweat test.

Interpretation	ECFS Guidelines [71]	Australian Guidelines [44,53]	The Manufacturer of the Nanoduct [72]
Normal (mmol/L)	<30	<40	<60
Positive (mmol/L)	≥60	>60	>80
Equivocal (mmol/L)	30–59	40–59 (infants 30–59)	60–80

**Table 2:** CF diagnostic criteria.

Study	Criteria	Reference
ECFS Standards of Care (2018)	Sweat chloride > 59 mmol/L and/or 2 CF-causing CFTR mutations in trans and at birth or clinical features, including but not restricted to diffuse bronchiectasis, positive sputum cultures for a CF-associated pathogen (especially *P. aeruginosa*), exocrine pancreatic insufficiency, salt loss syndrome, and obstructive azoospermia (males). Clinical signs suggestive of CF + equivocal ST + the presence of 0–1 mutations require follow-up in a CF clinic + performance of other tests. Clinical signs suggestive of CF + equivocal ST: DNA sequencing and NPD.	[71]
CF Foundation (2015)	Positive neonatal screening + 2 mutations or signs evocative of CF or meconial ileus + confirmation by sweat test. Without NBS + ST equivocal: NPD or measurement of intestinal current.	[29]
Australasian Guideline (2017)	Positive neonatal screening + the presence of 2 CFTR mutations. ST for confirmation diagnosis.	[81]
European Diagnostic Working Group (2006)	One/more phenotypic characteristics. Sweat test > 60 mmol/L. Pancreatic insufficiency/sufficiency. Severe evolution, with rapid progression of symptoms.	[143]
Royal College of Paediatrics ND Child Health (2017)	The identification of 2 CFTR mutations + the presence of clinical symptoms does not require confirmation by the sweat test. The neonatal screening program recommends performing the ST after positive neonatal screening in the presence of 2 CFTR mutations.	[142]
American Diagnostic Criteria (2003)	Highlights 2 CFTR mutations + increased chloride values >60 mmol/L in the ST.	[91]

## Data Availability

Not applicable.
